# The modified German subjective vitality scale (SVS-GM): Psychometric properties and application in daily life

**DOI:** 10.3389/fpsyg.2022.948906

**Published:** 2022-07-29

**Authors:** Laura Buchner, Günter Amesberger, Thomas Finkenzeller, Stephanie R. Moore, Sabine Würth

**Affiliations:** Department of Sport and Exercise Science, University of Salzburg, Salzburg, Austria

**Keywords:** subjective vitality, well-being, scale validation, experience sampling, diary study

## Abstract

Subjective vitality describes the positive feeling of experiencing physical and mental energy, which can lead to purposive actions, but no German instruments exist with action-oriented verbiage: This work supports the development and modification of already existing German Subjective Vitality Scales and provides further evidence for its psychometric properties. In a first step (*N* = 56) two modified (action-oriented) short-forms were developed. An extension of time perspectives (past, present, future) should also enrich the scale by enhancing the accuracy of self-reports. Study 1 (*N* = 183) then examined the psychometric properties for each time perspective. Study 2 (*N* = 27) was a 6-day diary study to identify the reliability of within- and between-person differences in vitality over time and working days with responses recorded three times per day. The exploratory factor analysis from study 1 revealed a three-factor solution with three items each. Test-retest reliability was moderate for the past and future time perspective and less stable for state subjective vitality. The modified German Subjective Vitality Scale (SVS-GM) showed divergent validity with fatigue, negative affect, and optimism, and convergent but distinguishable validity with life satisfaction, positive affect, and perceived self-efficacy. High reliability for daily vitality measures (with lower vitality rates in the morning) was found in study 2, but no substantial variation was found between working days and days off. The SVS-GM shows good psychometric properties in different settings and provides researchers with a 3-item (for cross-sectional or longitudinal studies) and 1-item (for short screenings) version to measure subjective vitality in German-speaking populations.

## Introduction

In the past decades, the concept of vitality has gained popularity in research that addresses indicators of subjective well-being (Guerin, 2012). Ryan and Frederick (1997) describe subjective vitality as the “conscious experience of possessing energy and aliveness” (p.539). Since then, the definition of the construct became less precise (Lavrusheva, 2020). The scoping review by Lavrusheva (2020) discloses five main features of vitality which characterize it as subjective, positive, dynamic, adaptive, and affected by both physical and psychological energy. External circumstances, as well as physical factors and psychological dispositions, may influence the inner individual resource of vitality (Ryan and Frederick, 1997; Lavrusheva, 2020). Thus, somatic factors like physical health, sleep, or exercise can influence the subjective experience of vitality. In the same way, psychological factors such as mental health or autonomous motivation affect subjective vitality (Ryan and Frederick, 1997; Ryan and Deci, 2008). The individual feeling of inner energy can also serve as a personal resource to initiate action. As a consequence, people have more readiness for purposive action when being in vital states (Ryan and Deci, 2008; Lam et al., 2014). Further, the energizing effect of vitality on behavior can lead to better coping with life challenges (Benyamini et al., 2000; Ryan and Deci, 2008). Subjective vitality can therefore be considered a multifaceted construct that has the ability to affect action initiation.

The Subjective Vitality Scale (SVS) by Ryan and Frederick (1997) is one of the most widely used scales to measure trait and state subjective vitality in different cultures and languages. The original version consists of seven items and was validated in a series of studies related to depression, anxiety, self-actualization, self-determination, physical health, life satisfaction, positive and negative affect, and the Big Five personality traits (Ryan and Frederick, 1997). Numerous shortened versions now exist of the original SVS. A 6-item version was suggested by Bostic et al. (2000) who used structural equation modeling techniques to prove the model fit. A 6-item version was also recommended by Castillo et al. (2017) and Couto et al. (2017) in Spanish and Portuguese, respectively. All 6-item versions removed the only negatively worded item (*I don’t feel very energetic*). Furthermore, translations and validations in other languages showed that deleting item 5 (*I look forward to each new day*) leads to better goodness-of-fit, likely because this item may be more related to the construct of optimism (Kawabata et al., 2017) and positive affect rather than an energetic and vital experience (Bertrams et al., 2020). Consequently, a 5-item version is confirmed for Japanese and Singaporeans (Kawabata et al., 2017) and Germans (Goldbeck et al., 2019; Bertrams et al., 2020). Using item response theory modeling, Kokou-Kpolou and Park (2020) demonstrated that even a 4-item French SVS is a reliable and valid instrument.

In prior research, the SVS demonstrated good levels of reliability with estimates of Cronbach’s α usually exceeding 0.83 (e.g., Ryan and Frederick, 1997; Goldbeck et al., 2019; Bertrams et al., 2020). Further, trait subjective vitality had higher test-retest reliability over 2–3 weeks than state subjective vitality (Bertrams et al., 2020). This suggests a less stable state subjective vitality over time. Temporal differences in life satisfaction showed that the inclusion of time dimensions (past, present, and future perspectives) may lead to a more concrete assessment of self-reported life satisfaction (Pavot et al., 1998). Since judgments of the past may influence the present and future view (Sailer et al., 2014) and a positive outlook on the future can impact the ability to cope with current situations (Pavot et al., 1998), a three-dimensional subdivision to a past, present, and future time perspective may also be constructive for the SVS.

Several studies have thus far proven the sensitivity of the state SVS to daily psychological and physical changes. A significant positive relationship has been found between perceived autonomy, competence, and relatedness with daily well-being (the aggregated sum score of different well-being measures; Sheldon et al., 1996) and subjective vitality (Reis et al., 2016). Specifically, participants reported higher values of positive mood and vitality when feeling more autonomous and competent during the day. In the same studies, higher positive scores were reported on weekend days due to the experience of more volitional and self-selected activities. This is not surprising, since the fulfillment of autonomy, competence, and relatedness are important basic psychological needs in the theory of self-determination (Deci and Ryan, 1985). Consequently, the fulfillment of these needs enhances subjective vitality (Ryan and Deci, 2000, 2008). Further works suggest subjective vitality changes with decreasing energy during working days (Ryan et al., 2010; Benedetti et al., 2015). However, Smolders et al. (2013) reported a parabolic fluctuation of subjective vitality during a day. In their study, subjective vitality increases with a higher amount of light exposure and sleep quality but decreases with a higher experience of fatigue.

## Aims of the current work

The different SVS versions contain cultural and linguistic discrepancies in their psychometric properties. Goldbeck et al. (2019) and Bertrams et al. (2020) used backward-forward approaches to translate the original English SVS (Ryan and Frederick, 1997) to German. This approach may lead to misinterpretations of several item meanings. So far, the aspect of activation of physical and mental resources to initiate actions is not encompassed in the existing German SVS. However, since this aspect is an important component of subjective vitality, the purpose of the current study is to reconsider the wording of the existing SVS and to develop a modified German SVS (SVS-GM). Furthermore, an extension to a past and future time perspective may enrich the new SVS-GM, since focusing on specific time perspectives allows a more sensitive temporal self-assessment than only the state and trait versions (Pavot et al., 1998; Carrillo et al., 2018). Further, to our knowledge, a detailed psychometric analysis for the German SVS used in a daily diary context is missing. The goal of psychometric analysis for longitudinal measures is to determine how reliably between-person differences and within-person changes are measured (Bolger, 2013). In this context, minimizing the number of items in the SVS reduces participants’ overall burden in experience-sampling studies (Shrout and Lane, 2012). Even with five items, the existing German SVS would be quite expensive and time-consuming when used in such study designs. Hence, to address these issues, two modified German short-forms of the SVS with reduced items and increased comprehensibility (i.e., action-oriented wording) and with extensions to a past and future time perspective were developed (see Supplementary Material 1). The first study evaluated then the psychometric properties and the construct validity of the new developed 3-item and a 1-item SVS-GM (SVS-GM3/SVS-GM1). The goal of the second study was to determine their reliability in the context of an intensive longitudinal measurement. For both studies, the local ethics committee granted approval and participants gave their written informed consent before participation.

## Study 1

The first objective of study 1 was to evaluate the SVS-GM descriptively using item analysis methods (Moosbrugger and Kavela, 2012). Second, we aimed to explore the reliability of the SVS-GM. We hypothesized adequate levels of internal consistency comparable to the original and German counterparts for all subsamples (Hypothesis 1, H1). Test-retest reliability was assessed by repeating the measures of the SVS-GM 2–3 weeks later with part of the sample. Similar to the results of Bertrams et al. (2020), we expected higher test-retest reliability over 2–3 weeks for past and future vitality than for state vitality (Hypothesis 2, H2). Third, we investigated the factor structure of the SVS-GM3 by conducting an exploratory factor analysis (EFA). We expected a 3-dimensional factor solution based on the three given time perspectives (past, present, future) (Hypothesis 3, H3). Fourth, we examined divergent and convergent construct validity by comparing related measures to the SVS-GM. Since subjective vitality is often described as an indicator of well-being (Guerin, 2012; Longo et al., 2017), we chose the construct of subjective well-being, which is described by life satisfaction and the balance between positive and negative affect (Diener, 1984). We hypothesized a moderate positive correlation between life satisfaction, positive affect, and subjective vitality (convergent validity), and a low to moderate negative relationship between negative affect and subjective vitality (divergent validity) (Hypothesis 4, H4). Based on the assumption that the conscious experience of feeling vital and energized can lead to action initiation and goal-directed behavior (Ryan and Deci, 2008; Çelik, 2017), we also included measurements of perceived self-efficacy. Self-efficacy beliefs are personal convictions that help one cope successfully with difficult situations in life and are the central determinants underlying human behavior (Bandura, 1977). Current German SVS versions (Schmitt et al., 2017; Goldbeck et al., 2019) may correlate low to moderate with subjective vitality because they do not cover enough of the energetic meaning of subjective vitality to initiate action and support proactive behavior. For this reason, we assumed moderate to high correlations between the SVS-GM and perceived self-efficacy (Hypothesis 5, H5). We also included measures of optimism to test whether subjective vitality and optimism differed. We expected low to moderate positive correlations between these two constructs (Hypothesis 6, H6). Another divergent assessment we used for validity was perceived fatigue. Perceived fatigue describes a holistic, perceptual, and complex phenomenon in which physical and cognitive functions are impaired (Enoka and Duchateau, 2016; Micklewright et al., 2017). Therefore, we expected higher values of fatigue when people felt less vital (Hypothesis 7, H7). Lastly, we compared the SVS-GM3 and the SVS-GM1. We assumed a high degree of comparability (Hypothesis 8, H8).

### Materials and methods

#### Sample and procedure

Data were collected online from two cohorts *via* LimeSurvey questionnaires at a German-speaking University in Austria between November 2019 and April 2020. In total, 279 participants visited the cover page of the survey at time point one (T1) with a completion rate of 65.59%. In total 183 native German speakers (*n* = 105 female; *M* = 23.16 years, *SD* = 3.41, 59.56% Austrians, 36.63% Germans, 2.19% other nationality) represent the full sample included in analysis. To determine the stability of the SVS-GM, measurements were repeated 2–3 weeks later with part of the sample. At this second time point (T2), 129 participants visited the cover page of the online survey, and 46.51% of participants (*N* = 60) completed the questionnaire (*n* = 40 female; *M* = 23.46 years, *SD* = 3.35).

Different measures for construct validity were split between T1 and T2 two reduce participant’s burden (Table 1). Nevertheless, for construct validation it was important to have small discrepancies in sample sizes between different measures. Since lower attendance at T2 was expected, the order of the utilized measures between the two cohorts per time point were switched to counteract this. Descriptive characteristics (age, sex) did not differ between the two cohorts.

**TABLE 1:** Materials used in study 1 at time point one (T1) and time point two (T2).

Cohort	T1	T2
A	Subjective Vitality	Subjective Vitality
	Fatigue	Fatigue
	Satisfaction with Life	Perceived Self-efficacy
	Affect	Optimism and Pessimism
B	Subjective Vitality	Subjective Vitality
	Fatigue	Fatigue
	Perceived Self-efficacy	Satisfaction with Life
	Optimism and Pessimism	Affect

#### Measures

##### Subjective vitality

We administered the SVS-GM3 and the SVS-GM1 to measure individual’s subjective vitality with past, present, and future time perspectives (see Table 2 and Supplementary Material 1). Regarding the goal of study 2 (reliability as a daily diary measure), the original 7-point scale was expanded to 11 points (0 = *not true at all* to 10 = *totally true*) with the assumption that it would enhance the sensitivity to short-term changes in subjective vitality. Mean SVS-GM3 scores were calculated for each time perspective.

**TABLE 2:** Descriptive statistics, item difficulties (p_i_), corrected item-total correlations (r_it_), and factor loadings of the EFA with the SVS-GM.

SVS-GM Items		*M*	*SD*	Skew.	Kurt.	*p*_i_	*r*_it_	Factor Loadings		
								
								F1	F2	F3
**Past:** In the last 2–3 weeks …										
1.	… I felt alive and vital.	6.07	2.55	−0.56	−0.56	60.70	0.83	**0.82**	0.15	0.00
2.	… I was full of drive.	6.32	2.36	−0.42	−0.42	63.20	0.83	**0.86**	0.00	0.00
3.	… I had energy and spirit.	6.58	2.32	−0.65	−0.65	65.80	0.88	**0.96**	0.00	0.00
SI	… I felt vital, full of drive and spirited. *	6.02	2.41	−0.50	−0.50	60.20	–	–	–	–
**Present/State**: At this moment …										
4.	… I feel alive and vital.	5.97	2.40	−0.26	−0.26	59.70	0.87	0.00	**0.95**	0.00
5.	… I am full of drive.	5.66	2.59	−0.21	−0.21	56.60	0.84	0.00	**0.87**	0.00
6.	… I have energy and spirit.	6.36	2.51	−0.43	−0.43	63.60	0.88	0.00	**0.81**	0.14
SI	… I feel vital, full of drive and spirited. *	5.95	2.47	−0.28	−0.28	59.50	–	–	–	–
**Future:** When I think about what’s coming up in the next 2–3 weeks …										
7.	… I feel alive and vital.	6.91	2.30	−0.81	−0.81	69.10	0.91	0.00	0.00	**0.90**
8.	… I am full of drive.	6.97	2.24	−0.76	−0.76	69.70	0.85	0.00	0.00	**0.88**
9.	… I have energy and spirit.	7.18	2.25	−0.79	−0.79	71.80	0.91	0.00	0.00	**0.95**
SI	… I feel vital, full of drive and spirited. *	6.68	2.31	−0.62	−0.62	66.80	–	–	–	–

N = 183, *SI = Single-item, Range = 0–10, Skew., Skewness; Kurt., Kurtosis.

EFA, Exploratory Factor Analysis, estimation method for the SVS-GM3 (9 items) was maximum likelihood with oblique rotation.

Factor loadings above 0.30 are in bold.

SVS-GM, 1-item and 3-item modified German Subjective Vitality Scale.

##### Fatigue

The Rate of Fatigue (ROF) is a 1-item scale capable of tracking perceptions of fatigue as a global, quantitative measure across various settings (e.g., during daily living, physical activity, or recovery; Micklewright et al., 2017). Bilingual native speakers translated the original English version into German by a backward-forward-backward approach. An interdisciplinary team of exercise and psychology scientists then discussed the applicability of the German wording in different settings. Perceived fatigue was assessed on an 11-point Likert scale ranging from 0 = *not fatigued at all*/*überhaupt nicht erschöpft* to 10 = *total fatigue and exhaustion—nothing left*/*total erschöpft und entkräftet—nichts geht mehr* (following Micklewright et al., 2017). Participants also completed the scale in the three different time perspectives (past, present, future), similar to the SVS-GM.

##### Affect

The original English Positive and Negative Affect Schedule (PANAS) was developed by Watson et al. (1988). In the current study, we included the reliable and valid German PANAS by Krohne et al. (1996). Each measures positive and negative affect on scales from 1 = *not at all* to 5 = *extremely* through ten items. Mean scores of each dimension describe low or high values of positive or negative affect. Importantly, positive and negative affect are two independent dimensions rather than bipolar dimensions (Watson et al., 1988; Krohne et al., 1996). Positive affect describes the amount of enthusiasm, action, and wakefulness a person exhibits. Negative affect is related to negative feelings like irritability, nervousness, or anxiety, and is often correlated with health problems (Watson et al., 1988). Participants also completed the PANAS related to the three time perspectives (past, present, future) of the SVS-GM. Internal consistency of positive affect ranged from α = *0.87* (past), 0.91 (present) to 0.89 (future) and of negative affect from α = 0.80 (past), 0.84 (present) to 0.86 (future).

##### Satisfaction with life

The satisfaction with life scale (SWLS) consists of five items that are answered on a 7-point Likert scale from 1 = *disagree* to 7 = *agree*. The resulting SWLS score is the sum of the five items and indicates low or high satisfaction with life. We applied the German version by Schumacher (2003), who translated the original English version (Diener et al., 1985). Differing instructions prompted participants to evaluate their satisfaction with life related to the three time perspectives of the SVS-GM. Cronbach’s α was 0.86 for all three time perspectives.

##### Perceived self-efficacy

The perceived self-efficacy scale (SWE) by Schwarzer and Jerusalem (1999) measures the subject’s conviction for mastering a difficult situation (where success is attributed to one’s competence). The scale consists of ten items rated on a 4-point Likert scale with 1 = *disagree* to 4 = *agree*. The sum of each answer describes the total score, which can range from 10 to 40. Cronbach’s α was 0.81.

##### Optimism and pessimism

The revised Life-Orientation Test (LOT-R; Glaesmer et al., 2008) is a two-dimensional, 10-item scale that measures optimism and pessimism and refers to an individual’s positive or negative outcome expectations. Both dimensions can vary independently. For example, a lower range of optimism is not necessarily associated with an increased range of pessimism. Three respective items assess optimistic and pessimistic tendencies. The remaining four items are included as filler items and are not relevant for further analysis. In this study, we utilized the original 5-point Likert scale to score the responses (1 = *disagree* to 5 = *agree*). The total LOT-R score results from the sum of optimism items and recoded pessimism items. Cronbach’s α coefficients confirmed internal consistency of 0.57 for optimism, 0.64 for pessimism, and 0.74 for the total scale.

### Statistical analysis

Using item analysis methods (Moosbrugger and Kavela, 2012), the SVS-GM was first evaluated descriptively. Internal consistency was assessed by Cronbach’s α. Test-retest correlations of the SVS-GM within participants at T1 and T2 were calculated to determine test-retest reliability over 2–3 weeks. To extract the number of relevant factors of the SVS-GM3, we conducted parallel analysis over all three time perspectives (9 items) using the full dataset from T1. Following Schmitt’s recommendations (2011), we applied exploratory factor analysis (*EFA*) with a maximum likelihood estimation method and oblique rotation to explore the loadings of each item. The maximum likelihood procedure provides standard errors and is an appropriate estimation method for multivariate normality considerations. Oblique rotation allows larger cross-loadings between factors. Since we expect correlations between the assumed vitality factors, direct oblimin rotation was used to produce a realistic and statistically sound factor structure (Schmitt, 2011; Field, 2018).

For construct validity, we investigated the PANAS and the SWLS from cohort A at T1 and from cohort B at T2 (total *N* = 143). SWE and LOT-R were assessed from cohort A at T2 and cohort B at T1 (total *N* = 100). In addition, the correlation between SVS-GM and ROF from both samples at T1 will be presented. We also calculated Pearson correlation coefficients between the SVS-GM3 mean score and the SVS-GM1 for each time perspective from T1. This way, similarities between these two short forms could be identified.

All statistical analysis was carried out in R (R Development Core Team, 2021). EFA and reliability calculations were performed with the R package *psych*, Version 2.0.12 (Revelle, 2020). Descriptive statistics are presented first, followed by the psychometric properties, and the results of reliabilities, EFA, and construct validation.

### Results

#### Descriptive statistics and reliabilities

In the current sample of study 1 at T1, SVS-GM3 mean scores ranged from *M* = 6.00–7.02 with slight skewness and kurtosis. Item difficulties were moderate, and all corrected item-total correlations were high (*r*_it_ > 0.83, Table 2). Cronbach’s α for the overall SVS-GM3 was high (α = 0.94), as were those for the subscales of the past (α = 0.93), present (α = 0.93) and future (α = 0.95). Furthermore, we examined the temporal stability of the SVS-GM in a subsample at T2. Test-retest reliability were moderate to high for all three time perspectives of the SVS-GM3 (*r*_tt_past_ = 0.55, *r*_tt_present_ = 0.42, *r*_tt_future_ = 0.54; *p* < 0.001) and low to high for the SVS-GM1 (*r*_tt_past_ = 0.53, *r*_tt_present_ = 0.27, *r*_tt_future_ = 0.47; *p* < 0.001).

#### Factorial structure

An EFA with oblique rotation (direct oblimin) was conducted on the overall SVS-GM3 (9 items) measured during T1. The Kaiser-Meyer-Olkin Measure of Sampling Adequacy verified the suitability of factor analysis (KMO ≥ 0.85) and Bartlett’s test of sphericity was significant (*p* < 0.001). Subsequent parallel analysis revealed a three-factor structure. All items of the three time perspectives loaded strongly on a corresponding factor with very small cross-loadings and explained a total variance of 83.6%. Table 2 shows the factor loadings for each item after oblimin rotation.

#### Construct validation

All correlations with the SVS-GM can be seen in Table 3. Correlations between all other variables can be found in the Supplementary Material 2. The SVS-GM was highly correlated with positive affect. Correlations with negative affect were negative and low to moderate. The SVS-GM also correlated highly with life satisfaction. According to these results, the SVS-GM differs from subjective well-being. As expected, the SVS-GM was also negatively correlated with ROF. A strong positive correlation was demonstrated between state SVS-GM and perceived self-efficacy, whereas a low correlation between state the SVS-GM and optimism was observed. The mean SVS-GM3 scores and SVS-GM1 were highly correlated (*r*_past_ = 0.84, *r_present_* = 0.79, *r*_future_ = 0.80; *p* < 0.001).

**TABLE 3:** Descriptive statistics and correlations with the SVS-GM3 and SVS-GM1 of the applied measures in study 1.

Measure	Time perspective	*N*	*M*	*SD*	Range	Correlation coefficient	
						
						SVS-GM3	SVS-GM1
Positive affect	Past	143	3.24	0.65	1–5	0.70**	0.71**
	Present	143	2.81	0.83	1–5	0.67**	0.72**
	Future	143	3.45	0.67	1–5	0.67**	0.59**
Negative affect	Past	143	1.94	0.58	1–5	−0.47**	−0.39**
	Present	143	1.45	0.51	1–5	−0.22**	−0.21*
	Future	143	1.60	0.54	1–5	−0.47**	−0.42**
Satisfaction with life	Past	143	23.58	5.81	1–7	0.64**	0.61**
	Present	143	24.53	5.85	1–7	0.49**	0.41**
	Future	143	25.17	5.29	1–7	0.55**	0.46**
Fatigue	Past	183	4.37	2.33	0–10	−0.59**	−0.59**
	Present	183	4.07	2.63	0–10	−0.51**	−0.52**
	Future	183	3.86	2.54	0–10	−0.39**	−0.43**
Self-efficacy	Trait	100	30.11	3.64	10–40	0.50**	0.45**
Optimism	Trait	100	22.56	3.62	6–30	0.26**	0.17

* indicates *p* < 0.05. ** indicates *p* < 0.01.

SVS-GM3, 3-item modified German Subjective Vitality Scale; SVS-GM1, 1-item modified German Subjective Vitality Scale.

### Discussion (study 1)

Overall, the vitality responses tended to be on the upper half of the scale (negative skewness). Such positive trends are typical for measures of well-being in healthy samples (Ryan and Frederick, 1997). According to Mummendey and Grau (2014), moderate item difficulties in this study indicate that the items can be used to differentiate persons with high and low vitality experiences. The item-total correlations were quite high, suggesting that items respond similarly to the different time perspectives (Moosbrugger and Kavela, 2012). Furthermore, the results are in line with the item-total correlations from the German SVS reported by Goldbeck et al. (2019).

Cronbach’s alphas are comparable to national (Goldbeck et al., 2019; Bertrams et al., 2020) and international equivalents (Martela and Ryan, 2016) showing sufficient reliability (H1). Test-retest reliability (2–3 weeks apart) was higher for the past and future perspective than for the state SVS-GM. These findings are in line with results from Bertrams et al. (2020), who also reported lower state test-retest correlations 3 weeks apart compared to trait dimensions. Test-retest reliability in other studies lay between *r*_tt_ = 0.44 for trait vitality (Castillo et al., 2017; Kawabata et al., 2017) and *r*_tt_ = 0.63 for vitality ratings of the past month (Ryan and Frederick, 1997). Lower state test-retest coefficients display a higher degree of changeability and therefore, a less stable state assessment. Otherwise, past and future dimensions were moderately stable assessments over the course of 2–3 weeks (H2). Thus, the time perspective extension leads to a focus on specific time periods and allows a more accurate assessment of subjective vitality.

The EFA resulted in a three-factor solution that also supports the application of time dimensionality in the SVS-GM. Each of the factors represented a corresponding time perspective (past, present, future) and thus demonstrates the factorial validity of the SVS-GM3 (H3). The clear 3-factor solution supports the notion that a temporal distinction of subjective vitality is helpful to enhance the accuracy of self-reports.

Construct validity was established by comparing the SVS-GM with convergent and divergent constructs. Our findings are in line with previous comparisons to positive affect (Martela and Ryan, 2016; Bertrams et al., 2020), negative affect (Ryan and Frederick, 1997; Bertrams et al., 2020), satisfaction with life (Ryan and Frederick, 1997; Castillo et al., 2017; Kawabata et al., 2017; Çelik, 2017; Goldbeck et al., 2019; Bertrams et al., 2020), and fatigue (Ryan and Frederick, 1997; Longo et al., 2017). Overall, the correlations show that the SVS-GM is related similarly to the important constructs used in other studies (H4, H7). One goal of the current study was to more precisely define items that represent an action-oriented behavior. For this reason, the construct of perceived self-efficacy was used to validate the SVS-GM. Goldbeck et al. (2019) supported a moderate correlation between perceived self-efficacy and the German trait version of the SVS and Schmitt et al. (2017) found low correlations. The results of the current study agree and support a moderate correlation between SWE and future SVS-GM, and a strong correlation of SWE with past and state SVS-GM. The lower correlation for the future time perspective may be explained by the different instructions; while participants rated their subjective vitality with three different time perspectives, perceived self-efficacy is not inherently time-specific. Personal memories influence ratings of past and present time perspectives and are closely related to trait dimensions like perceived self-efficacy rather than future time perspectives (Karniol and Ross, 1996). Thus, compared to Goldbeck et al. (2019), the larger correlation between SVS-GM and SWE in the present sample indicates that the new wording is more effective at describing the feeling of intrinsic motivation and goal orientation (H5). Furthermore, the SVS-GM can be differentiated from optimism (H6), indicated by a low correlation between LOT-R and SVS-GM. Comparable findings (Huppert and So, 2013), and also higher correlations, between optimism and subjective vitality (Longo et al., 2017) are found in the literature. Optimism and generalized self-efficacy beliefs are said to be stable personality characteristics (Schwarzer, 1994; Glaesmer et al., 2008). The reinforcement of a person’s belief in their resources and capabilities to successfully complete a task will lead to the formation of habits. This higher perceived self-efficacy, in the form of high personal conviction, is very important for action initiation in general and is based on the attitude of a person. Moreover, the optimistic personal attitude (i.e., belief in positive outcomes) is relevant for coping with stressors in daily life (Egger, 2015). Thus, the results of the construct validation with SWE and LOT-R illustrate a more action-oriented (rather than attitude-orientated) understanding of the SVS-GM.

The distinction from positive and negative affect, life satisfaction, fatigue, perceived self-efficacy, and optimism demonstrate that the SVS-GM is an appropriate and relevant instrument to measure subjective vitality efficiently. Both, the SVS-GM3 and the SVS-GM1 exhibit similar convergent and divergent relationships with the presented constructs. Additionally, high correlations between the SVS-GM3 and SVS-GM1 give researchers the possibility to use one of the presented versions without major loss of reliability (H8).

## Study 2

The goal of study 2 was to evaluate the reliability of within-person changes and between-person differences in everyday life in a 6-days electronic diary study. Using multilevel confirmatory factor analysis (Geldhof et al., 2014), Schmitt et al. (2017) proved the reliability of a 3-item SVS version on the between-person level (α = 0.97) and within-person level (α = 0.93) in a daily diary study. We assumed similar results in our sample based on psychometric analysis for longitudinal measures (Bolger, 2013) including a detailed method of variance decomposition following generalizability theory (GT) (Cronbach et al., 1972; Shrout and Lane, 2012) (Hypothesis 9, H9). Furthermore, we were interested in daily fluctuation and predictors of subjective vitality (time of the day, workday vs. day off, and fatigue). Due to previous study results (Ryan et al., 2010; Smolders et al., 2013; Benedetti et al., 2015), we expected daily and day type-dependent variations in subjective vitality at both the between- and within-person level in the present sample (Hypothesis 10, H10).

### Materials and methods

#### Sample and procedure

The sample was recruited by e-mail at the university and by word of mouth communication of several research assistants. In total, 28 young and healthy German-speaking females participated in a six-day diary study. Due to technical malfunctions, the data of one participant was lost. Thus, data from 27 females could be analyzed (*M*_age_ = 23.59 years, *SD*_age_ = 3.13). For this study, a smartphone diary App was designed for Android Version 6.0 and higher. Therefore, twenty-six participants used their personal smartphone for data collection. One person was provided an Android smartphone that was used only for data collection. This way, participants were able to answer the diary App questions in their natural surroundings, and data were anonymously stored on a backend server. Push reminders (acoustic or silent) were sent out in an interval-contingent design three times per day (morning: 7:00–11:00 am; noon: 12:30–3:30 pm; evening: 6:30–9:30 pm) over the six full days. If the diary was not fully completed after the first reminder, participants received follow-up reminders every 30 min (5 maximum). The maximum number of responses possible was 486 (27 participants × three daily assessments × 6 days). In the present study, the compliance rate was 92.3% (449 observations).

#### Measures

At each of the response times, participants rated the state SVS-GM3, SVS-GM1 (Table 2) and ROF (Micklewright et al., 2017) using a visual slider. After moving the slider from 0 to 10, the chosen value was clearly presented before participants proceeded with the next question.

### Statistical analysis

To assess the reliability of the SVS-GM as a daily diary measure, we followed the recommendation by Cranford et al. (2006) who describe the applications of GT (Cronbach et al., 1972). GT is a powerful psychometric method that decomposes the variance of daily measures into item, time, person, and their interactions combined with residuals (Shavelson et al., 1989). This is accomplished using a three-way, crossed random effects ANOVA. The relevant variance component estimates were subsequently used to calculate the reliability of between-person differences and within-person change. Calculations of different reliability coefficients evaluate the reliability of a scale when used in different study designs (Cranford et al., 2006; Shrout and Lane, 2012; Bolger, 2013).

Finally, we conducted three additional multilevel model analyses to examine daily and weekly fluctuations in subjective vitality and its relationship with fatigue. Observations (Level 1) were nested within participants (Level 2) and the ICC explained 46% of the between- and 54% of the within-person variance, suggesting the appropriateness of multilevel modeling. In our first model, we explored the influence of the diary time of day (i.e., morning/noon/night), the type of day (reported workday vs. day off), and ROF on subjective vitality. With the SVS-GM3 mean score as the dependent variable and time of day, type of day, and person-mean centered ROF as independent variables, a random intercept model was significantly better than the null model (logLikelihood: −704.22, *p* < 0.001). Further, a random slope model for ROF fitted the data better than the random intercept model (logLikelihood: −699.46, *p* = 0.009). In a second model, the same calculations were done with the SVS-GM1 as the criterion measure. The goal of this was to prove the sensitivity of the SVS-GM1 in a daily diary context. Lastly, in a third model, the relationship between the SVS-GM3 (dependent variable) and the person-mean centered SVS-GM1 (independent variable) was investigated. Calculations were done in R (R Development Core Team, 2021) using the *psych* package for GT applications (tutorial by Revelle and Wilt, 2019). Multilevel analysis were conducted with the lme4 package (Bates et al., 2015).

### Results

Table 4 shows the results of the decomposition of variance for the SVS-GM3. The largest source of variance was the between-person variation ($\sigma_{\text{PERSON}}^{2}$). Thus, some subjects had consistently higher vitality rates than others across time and items. The small amount of variance in time ($\sigma_{\text{TIME}}^{2}$) explained a relatively small variation in response rates across observations. Similarly, the very small between-item variance ($\sigma_{\text{ITEM}}^{2}$) and time by item variance ($\sigma_{\mathrm{TIME}*\mathrm{ITEM}}^{2}$) indicated that all three items were rated consistently within the dimension in general and over time. A high variance of the interaction between person and observation ($\sigma_{\mathrm{PERSON}*\mathrm{TIME}}^{2}$), supported that subjects differed in their response rates over time. The variance categorized as an error ($\sigma_{\text{ERROR}}^{2}$) describes the amount of variance of unusual responses of time points for persons and items. The different estimations of generalizability coefficients indicate substantial reliability between-person differences for the SVS-GM3 when measured over a fixed (R_KF_) or random (R_KR_) period as well as for measures taken on a single fixed day (R_1F_). The reliability of a randomly selected day (R_1R_) displays the coefficient when researchers are interested in measuring between-person differences with data collected on a different day. Of special interest is the coefficient to assess the within-person change over time (R_C_). The substantial value indicates that systematic change in vitality can be measured reliably with the SVS-GM3.

**TABLE 4:** Variance decomposition of the state SVS-GM3 and estimates of between- and within-person reliability.

Variance component	SVS-GM3	%
$\sigma_{\text{PERSON}}^{2}$	1.72	40.5
$\sigma_{\text{TIME}}^{2}$	0.14	3.3
$\sigma_{\text{ITEM}}^{2}$	0.06	1.5
$\sigma_{\mathrm{PERSON}*\mathrm{TIME}}^{2}$	1.69	39.6
$\sigma_{\mathrm{PERSON}*\mathrm{ITEM}}^{2}$	0.06	1.3
$\sigma_{\mathrm{TIME}*\mathrm{ITEM}}^{2}$	0.03	0.7
$\sigma_{\text{ERROR}}^{2}$	0.56	13.1
Total	4.27	100.0
R_KF_	0.99	
R_KR_	0.94	
R_1F_	0.90	
R_1R_	0.46	
R_c_	0.90	

R_KF_, Reliability coefficient of a fixed diary period; R_KR_, Reliability coefficient of different diary periods; R_1F_, Reliability coefficient of measures taken on a fixed day; R_1R_, Reliability coefficient of measures taken on a randomly selected day; R_C_, Within-person reliability coefficient over time; SMS-GM3, 3-item modified German Subjective Vitality Scale.

**FIGURE 1:**
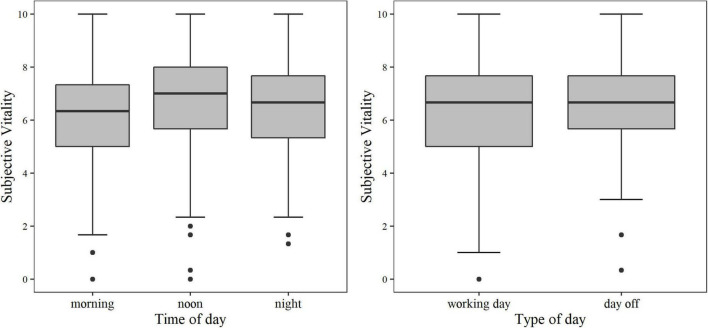
Subjective Vitality distribution between different times of day (left) and type of day (right).

**FIGURE 2:**
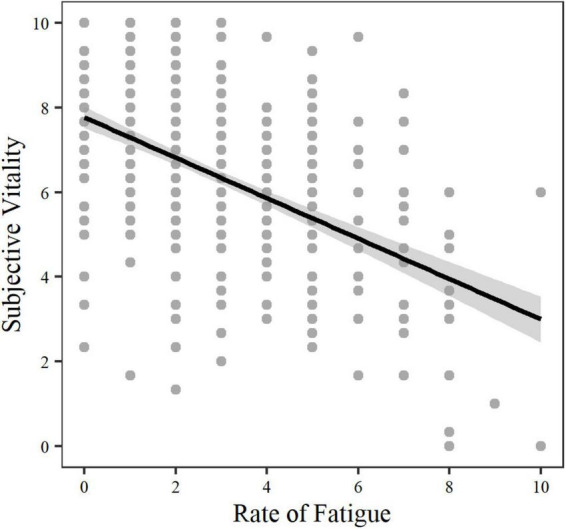
Subjective Vitality predicted by Rate of Fatigue overall measurement points.

The results of the multilevel model analysis are presented in Table 5 and Figures 1, 2. Subjective vitality was lowest in the morning when compared to noon and evening (*p* < 0.001). Subjective vitality was only significantly different on working days compared to days off, in simple model calculations without ROF as a predictor. Furthermore, ROF negatively predicted subjective vitality (*p* < 0.001) with a different influence between subjects (slope). Model 1 demonstrates within-person variation and between-person differences of the SVS-GM3 during the diary study. Similar results are confirmed with the SVS-GM1 as the dependent variable (Model 2; see Supplementary Material 3). When predicting the SVS-GM3 mean score by the SVS-GM1, the two scale versions were almost identical [Model 3: *b* = 0.94, *t*(444) = 53.94, *p* < 0.001].

**TABLE 5:** Model parameters for the multilevel analysis with the mean score of the SVS-GM3 as the dependent measure (Model 1).

Parameter	Estimate (SE)	*p*	95% CI	
			
			Lower	Upper
**Fixed effects**				
Intercept	5.94 (0.28)	< 0.001	5.39	6.49
Noon vitality	0.51 (0.12)	< 0.001	0.27	0.75
Evening vitality	0.63 (0.12)	< 0.001	0.38	0.87
Day off	0.18 (0.11)	0.112	−0.04	0.40
ROF	−0.46 (0.04)	< 0.001	−0.54	−0.39
**Random effects**				
**Level 2 (between-person)**				
Intercept	1.81		1.02	1.78
ROF (Slope)	0.02		0.06	0.21
**Level 1 (within-person)**				
Residual	1.05		0.95	1.09

Standard errors are in parentheses.

All p values in this table are two-tailed.

SMS-GM3, 3-item modified German Subjective Vitality Scale.

The reference category for the variables noon vitality and evening vitality is morning vitality.

### Discussion (study 2)

The goal of study 2 was to explore the psychometric properties of the SVS-GM3 in an experience-sampling design. The variance decomposition and reliability coefficients prove that the SVS-GM3 is a valid instrument to measure between-person differences and within-person changes in daily life (H9). Multilevel analyses revealed that subjective vitality was lowest in the morning and increased until noon, after which it stayed relatively constant until evening (H10). Lower morning vitality responses were also reported in other studies (Smolders et al., 2018; Cangiano et al., 2019). Our results also show that the type of day (reported workday vs. day off) is not a predictor of subjective vitality (H10). Although, simple models without ROF as a predictor showed a significant negative impact of working days on subjective vitality [*b* = −0.303, *t*(433.5294) = −2.03, *p* = 0.043]. Within a more complex model, however, this significant effect disappears. Similarly, Kukita et al. (2020) explored the influence of different activities (rest, work, study, play) on momentary affect and found that once other measures (like autonomy) are included and add complexity to the model, the significant effect of the different activities on momentary affect was lost. In the present sample, the ratio of working days to free days was approximately 50% but significant differences between day types are not displayed in the final model. One reason for this distribution could be the high number of students (75%) included in our sample. Typically, students do not conform to a 5-day work week. Moreover, some data collection fell into the time of semester holiday. Thus, some participants reported only working days or days off during the period of data collection. Oppositely, previous studies highlighted an increase in vitality on weekend days due to a higher fulfillment of basic needs (Ryan et al., 2010; Weigelt et al., 2021). We assume that due to the high rate of days off in our sample, effects like *Thank Goodness it’s Friday* or *Blue Monday* (Stone et al., 2012) are not strong enough to be influential in a more complex model.

Experience-sampling studies that focused on the relationship between vitality and fatigue demonstrated that vitality decreases with an increase in experienced fatigue (Smolders et al., 2013; Weigelt et al., 2021). Results in the present study are in line with previous research related to this topic (H7).

One of the main goals of this study was to develop a 3-item and 1-item short form of the existing SVS to reduce participants’ burden in experience sampling studies. Both short-forms show consistent results in their characteristics related to multilevel results, therefore we deem both short-forms appropriate for future analysis. However, Shrout and Lane (2012) recommend that a minimum of three daily items per scale should be provided to reduce measurement errors in experience-sampling studies. Therefore, we recommend using the SVS-GM3 for elaborated statistical analyses in cross-sectional or longitudinal studies and the SVS-GM1 version only for short screenings.

## Overall discussion

The goal of the different studies was to modify the German SVS in such a manner that it covers more precisely the energizing aspect of action initiation. Especially, the results from study 2 suggest that correlations with perceived self-efficacy are higher compared to other German translations (Schmitt et al., 2017; Goldbeck et al., 2019). Nevertheless, a closer inspection of the coefficients (Table 3) illustrates lower coefficients of the SVS-GM1 compared to SVS-GM3 with all other constructs (except ROF). A reason for the apparently lower validity of the SVS-GM1 is probably the lower number of items. In general, it is known that subjective/psychological constructs reach higher precision by increasing the number of items per dimension or scale because more items per scale can compensate for measurement errors (Mossbrugger and Kelava, 2012). This seems also true for the developed SVS-GM1 and SVS-GM3. The identical (past) and slightly higher (present, future) correlation coefficients between ROF and SVS-GM1 compared to SVS-GM3 may be a result of the comparison of two 1-item scales. Both the ROF and SVS-GM1 are measured with a single item which may cause the slightly higher correlations.

A greater reduction in measurement errors may also be seen in the test-retest reliability of the SVS-GM3. The low test-retest reliability of the state SVS-GM1 is not satisfactory when compared to highly controlled analyses. We believe that mainly uncontrollable situational dependencies (environment, time of day, etc.) could lead to measurement inaccuracies. These influences seem to be better buffered by the SVS-GM3 than by the SVS-GM1. However, we do not assume test-retest reliability in the narrow sense. Rather, we wanted to map the dynamics of the construct and the distinctions of different time perspectives. For instance, past and future SVS show higher test-retest reliability than present SVS for both the SVS-GM1 and SVS-GM3. This is in line with the fundamentals of trait-state distinctions that describe the stability and variability of human attitudes and behavior over time. Whereby it is assumed that traits remain as rather stable dispositions over time, states tend to reflect the person’s variability to circumstances (Hamaker et al., 2007). As a consequence, higher stability can be observed in trait subjective vitality (past, future), and more variability in state subjective vitality. Additionally, higher correlation coefficients are also observed between trait subjective vitality and quite stable attitudes like perceived self-efficacy and optimism compared to state subjective vitality (Supplementary Material 2). In other words, the extension of different time perspectives opens new possibilities for the validity and reliability of the SVS-GM and underlines the importance of this differentiation. Especially for purposive actions, the evaluation of different time perspectives is relevant for decision making and action execution (Boniwell et al., 2010). Moreover, people feel better in the present and look forward to a more positive future when they remember a happier and less gloomy past (Sailer et al., 2014). Thus, the higher values of reliability and validity in the SVS-GM3 lead us to the recommendation of using the 3-item scale mainly in longitudinal and complex studies, as this version seems to forgive situational discrepancies more easily. However, for short screenings with simple study designs and time restrictions, the SVS-GM1 is an appropriate and valid measurement to assess subjective vitality.

## Conclusion

The findings of the two studies support the development of a reliable, valid, and economic instrument that measures subjective vitality in a German-speaking population. The adaptation of a past and a future time perspective allows for a more specific classification of vitality. Construct validation supports the idea that subjective vitality is correlated in a fair to good range with divergent and convergent measures. Reliability coefficients, in the context of experience-sampling studies, demonstrate that the SVS-GM3 is a sensitive measure to detect not only between-person differences but also within-person changes over time. The results provide further evidence for the validity of the SVS-GM in a relatively young sample, which extends the findings from other research on subjective vitality that were also conducted with young samples (e.g., Martela and Ryan, 2016; Kawabata et al., 2017; Goldbeck et al., 2019; Bertrams et al., 2020). On the other hand, the transfer of the results to a generalized population is limited. The samples collected in the current studies were between the ages of 18 and 30 years with little educational and occupational diversity. Moreover, study 2 only consists of females with a high proportion of students. Despite these limitations, the current validation of SVS-GM can make the combination of self-reported and objective measures (e.g., accelerometry, morning heart rate variability) more practical. Thus, future research may validate the SVS-GM with more diverse population samples and further encourage the investigation of subjective and objective measures. Ultimately, the SVS-GM3 and SVS-GM1 are both acceptable measures for research and practice. The use of the SVS-GM3 is more suitable for cross-sectional or longitudinal studies, whereas the SVS-GM1 is more for practice and short screenings. Therefore, we encourage future research to explore the construct of subjective vitality in different settings and populations.

## Data availability statement

The datasets presented in this article are not readily available because of sharing agreements in the funded project. Requests to access the datasets should be directed to laura.buchner@plus.ac.at.

## Ethics statement

The studies involving human participants were reviewed and approved by the Ethics Committee of the Paris Lodron University Salzburg (EK-GZ: 33/2019). The patients/participants provided their written informed consent to participate in this study.

## Author contributions

LB, GA, TF, and SW conceptualized the study and organized the data collection. LB was responsible for the formal analysis and writing the manuscript. SM reviewed the international English language standards in the manuscript. All authors discussed the statistical analysis, edited the manuscript, and approved the submitted version.

## Supplementary material

SUPPLEMENTARY MATERIAL 1 SM1_Buchner et al., SVS-GM The development of the modified German Subjective Vitality.

SUPPLEMENTARY MATERIAL 2 SM2_Buchner et al., SVS-GM Correlation Coefficients between Measures from Study 1.

SUPPLEMENTARY MATERIAL 3 SM3_Buchner et al., SVS-GM Multilevel Analysis with the SVS-GM1 as the criterion measure.
