# Supplementary material for: The modified German subjective vitality scale (SVS-GM): Psychometric properties and application in daily life
Source: Front Psychol. 2022 Jul 29;13:948906. doi: 10.3389/fpsyg.2022.948906 (PMC9374102; doi:10.3389/fpsyg.2022.948906)
Supplement: SUPPLEMENTARY MATERIAL 1 — SM1_Buchner et al., SVS-GM The development of the modified German Subjective Vitality. [file Table_1.pdf]

Supplementary Material 1

1 The development of the modified German Subjective Vitality Scale (SVS-GM)

None of the current German versions of vitality measures focus on the aspect of proactive behavior. Therefore, we reconsidered the wording of the existing German SVS items to a more action-oriented meaning of subjective vitality that also includes a past and future time perspective intending to enhance the accuracy of self-reports. In the following, a 3-item short form (SVS-GM3) is verified on a small sample for linguistic comprehension.

2 Methods

2.1 Development of the modified German SVS

On account of recent study results and discussions (Bertrams et al., 2020; Bostic et al., 2000; Ryan & Frederick, 1997), the selection of items for the current SVS-GM was based on content and linguistic-related aspects (Edwards, 1983). Items of the original SVS, its German counterpart, and its developed modified version are displayed in the Supplemantal Table 1. Since item 5 seems to be challenging on factor loadings and construct validity (Bertrams et al., 2020), we decided to reword this item to express a more action-related orientation. Therefore, we decided on a new item that combined items 5 and 3. The resulting new item was a typical German expression implying energetic ambition: *Ich bin voller Tatendrang*. *Tatendrang* is defined as the drive to be active and to accomplish something (Dudenredaktion, n.d.; Drang, sich zu betätigen, etwas zu leisten). The precise translation is difficult due to its linguistic and cultural meaning, however, the English expression *I am full of drive* is very close. The original items 1 and 4 were transferred without any changes. The remaining items 6 and 7 were not included in the SVS-GM because the attentional component of *alert and awake* is not part of the definition of subjective vitality. Moreover, they are also more related to mood (Watson et al., 1988) than to the alignment with eudaimonic well-being and in this context with subjective vitality (Ryan & Deci, 2001).

Supplementary Table 1

Items of the Original English Subjective Vitality Scale (Ryan & Frederick, 1997), their German Translation (Bertrams et al., 2020), and their Modified German Version Developed in the Current Study

Item No	English	German	German-modified
1	I feel alive and vital.	Ich fühle mich lebendig und vital.	Ich fühle mich lebendig und vital.
2	I don’t feel very energetic.		
3	Sometimes I am so alive I just want to burst.	Manchmal fühle ich mich so lebendig, dass ich platzen könnte.	Ich bin voller Tatendrang. **
4	I have Energy and Spirit.	Ich habe Energie und Lebensfreude.	Ich habe Energie und Lebensfreude.
5	I look forward to each new day.	Ich freue mich auf jeden neuen Tag. *	
6	I nearly always feel awake and alert.	Ich fühle mich nahezu immer aufmerksam und wach.	
7	I feel energized.	Ich fühle mich energiegeladen.	

Note. * The authors recommend omitting this item. ** Reconsidered wording by a combination of items 3 and 5 towards an action-oriented verbiage.

For the SVS-GM, the original 7-point scale was expanded to 11 points (0 = *not true at all* to 10 = *totally true*) with the assumption that it would enhance the sensitivity to short-term changes in

subjective vitality. As a result, the fluctuation of vitality across the day could be detected in a more precise way. To include the multiple time dimensions, subjects were instructed to rate the SVS-GM items from three time perspectives: (past) how they felt in the previous 2-3 weeks, (present) how they felt at that moment, and (future) how they thought they would feel in the next 2-3 weeks.

2.2 Sample and Procedure

Recruitment took place at a German-speaking university in Austria. Native German speakers were selected if they met the age criteria of 18-30 years. To test linguistic comprehensibility, data from 57 participants (n = 29 female; M = 24.25 years, SD = 3.73) were collected by a paper-and-pencil questionnaire followed by an in-person half-standardized interview in November 2019. The whole data collection procedure took about 5-10 min.

2.3 Measures

To measure the perceived subjective vitality of individuals the SVS-GM3 including the three different time perspectives (past, present, future) was used.

The half-standardized interview included questions about the comprehensibility of the instruction, items, and content. It was important to know if participants understood the semantic meaning of the combined construction of items 3 and 5. In particular, participants explained what the term *Tatendrang* meant to them.

3 Results

To most of the participants, the scales, instructions, and items were very clear and concise. Reports explaining the term *Tatendrang* were close to the definition of Duden (Dudenredaktion, n.d.). *Tatendrang* was often associated with motivation and the energized feeling to start voluntary tasks. Furthermore, the joy and desire to act were used in many explanations. However, the evaluation of participants' feedback highlighted missing general scale information and difficulties to rate future subjective vitality. The uncertainty was to estimate subjective vitality in the next 2-3 weeks, as this was difficult to predict. As a result, we extended the total scale instruction and improved the instructions for vitality items in the future perspectives. Further, a single item (SVS-GM1) was created, which combines expressions of the three SVS-GM3 items. The full instructions and scales of the SVS-GM are listed below.

The 3-item modified German Subjective Vitality Scale (SVS-GM3)

Nachfolgend möchten wir gerne von Ihnen wissen, wie Sie folgende Aussagen einschätzen. Bitte benutzen Sie die Skala von 0-10, um Ihre Zustimmung bzw. Ablehnung zu jeder Aussage zum Ausdruck zu bringen. Denken Sie nicht lange nach, sondern antworten Sie spontan!

**In den letzten 2-3 Wochen ...**	Trifft gar nicht zu									Trifft völlig zu	
	0	1	2	3	4	5	6	7	8	9	10
… fühlte ich mich lebendig und vital.	☐	☐	☐	☐	☐	☐	☐	☐	☐	☐	☐
… war ich voller Tatendrang.	☐	☐	☐	☐	☐	☐	☐	☐	☐	☐	☐
… hatte ich Energie und Lebensfreude.	☐	☐	☐	☐	☐	☐	☐	☐	☐	☐	☐

**In diesem Moment …**	Trifft gar nicht zu									Trifft völlig zu	
	0	1	2	3	4	5	6	7	8	9	10
… fühle ich mich lebendig und vital.	☐	☐	☐	☐	☐	☐	☐	☐	☐	☐	☐
… bin ich voller Tatendrang	☐	☐	☐	☐	☐	☐	☐	☐	☐	☐	☐
… habe ich Energie und Lebensfreude.	☐	☐	☐	☐	☐	☐	☐	☐	☐	☐	☐

**Wenn ich daran denke, was in den nächsten 2-3 Wochen auf mich zukommt, …**	Trifft gar nicht zu									Trifft völlig zu	
	0	1	2	3	4	5	6	7	8	9	10
… fühle ich mich lebendig und vital.	☐	☐	☐	☐	☐	☐	☐	☐	☐	☐	☐
… bin ich voller Tatendrang	☐	☐	☐	☐	☐	☐	☐	☐	☐	☐	☐
… habe ich Energie und Lebensfreude.	☐	☐	☐	☐	☐	☐	☐	☐	☐	☐	☐

The 1-item modified German Subjective Vitality Scale (SVS-GM1)

Nachfolgend möchten wir gerne von Ihnen wissen, wie Sie folgende Aussagen einschätzen. Bitte benutzen Sie die Skala von 0-10, um Ihre Zustimmung bzw. Ablehnung zu jeder Aussage zum Ausdruck zu bringen. Denken Sie nicht lange nach, sondern antworten Sie spontan!

	Trifft gar nicht zu									Trifft völlig zu	
	0	1	2	3	4	5	6	7	8	9	10
In den letzten 2-3 Wochen fühlte ich mich vital, voller Tatendrang und Lebensfreude.	☐	☐	☐	☐	☐	☐	☐	☐	☐	☐	☐
In diesem Moment fühle ich mich vital, voller Tatendrang und Lebensfreude.	☐	☐	☐	☐	☐	☐	☐	☐	☐	☐	☐
Wenn ich daran denke, was in den nächsten 2-3 Wochen auf mich zukommt, fühle ich mich vital, voller Tatendrang und Lebensfreude.	☐	☐	☐	☐	☐	☐	☐	☐	☐	☐	☐

4 References

Bertrams, A., Dyllick, T. H., Englert, C., & Krispenz, A. (2020). German Adaptation of the Subjective Vitality Scales (SVS-G). *Open Psychol.*, *2*(1), 57-75. https://doi.org/10.1515/psych-2020-0005

Bostic, T. J., McGartland Rubio, D., & Hood, M. (2000). A validation of the subjective vitality scale using structural equation modeling. *Soc. Indic. Res.*, *52*(3), 313-324. https://doi.org/10.1023/a:1007136110218

Dudenredaktion. (n.d.). Tatendrang. In *Duden online*. Retrieved 14.12.2021, from https://www.duden.de/node/179802/revision/418842

Edwards, A. L. (1983). *Techniques of attitude scale construction*. Appleton.

Ryan, R. M., & Deci, E. L. (2001). On happiness and human potentials: a review of research on hedonic and eudaimonic well-being. *Annu. Rev. Psychol.*, *52*, 141-166. https://doi.org/10.1146/annurev.psych.52.1.141

Ryan, R. M., & Frederick, C. (1997). On energy, personality, and health: subjective vitality as a dynamic reflection of well-being. *J. Pers.*, *65*(3), 529-565. https://doi.org/10.1111/j.1467-6494.1997.tb00326.x

Watson, D., Clark, L. A., & Tellegen, A. (1988). Development and validation of brief measures of positive and negative affect: the PANAS scales. *J. Pers. Soc. Psychol.*, *54*(6), 1063-1070. https://doi.org/10.1037//0022-3514.54.6.1063
