# Supplementary material for: The modified German subjective vitality scale (SVS-GM): Psychometric properties and application in daily life
Source: Front Psychol. 2022 Jul 29;13:948906. doi: 10.3389/fpsyg.2022.948906 (PMC9374102; doi:10.3389/fpsyg.2022.948906)
Supplement: SUPPLEMENTARY MATERIAL 2 — SM2_Buchner et al., SVS-GM Correlation Coefficients between Measures from Study 1. [file Table_2.pdf]

Supplementary Material 2

1 Correlation Coefficients between Measures from Study 1

Supplementary Table 2

Means (M), standard deviations (SD), and Pearson product-moment correlation coefficients between the SVS-GM3, SVS-GM1, and ROF

Variable		M	SD	1	2	3	4	5	6	7	8
SVS-GM3	past (1)	6.32	2.25								
	present (2)	6.00	2.35	.68** [.59, .75]							
	future (3)	7.02	2.15	.59** [.49, .68]	.63** [.54, .71]						
SVS-GM1	past (4)	6.02	2.41	.84** [.79, .88]	.60** [.50, .68]	.51** [.39, .61]					
	present (5)	5.95	2.47	.57** [.46, .66]	.79** [.73, .84]	.52** [.41, .62]	.65** [.55, .72]				
	future (6)	6.68	2.31	.54** [.43, .64]	.61** [.52, .70]	.80** [.74, .85]	.54** [.43, .63]	.58** [.47, .67]			
ROF	past (7)	4.37	2.33	-.59** [-.68, -.49]	-.35** [-.47, -.21]	-.31** [-.44, -.17]	-.59** [-.68, -.48]	-.36** [-.48, -.23]	-.33** [-.45, -.19]		
	present (8)	4.07	2.63	-.30** [-.43, -.16]	-.51** [-.61, -.40]	-.23** [-.36, -.09]	-.29** [-.42, -.15]	-.52** [-.62, -.41]	-.33** [-.45, -.19]	.44** [.32, .55]	
	future (9)	3.86	2.54	-.29** [-.41, -.15]	-.30** [-.43, -.17]	-.39** [-.51, -.26]	-.26** [-.39, -.12]	-.29** [-.41, -.15]	-.43** [-.54, -.30]	.46** [.34, .57]	.46** [.33, .56]

Note. N = 183

Values in square brackets indicate the 95% confidence interval for each correlation. The confidence interval is a plausible range of population correlations that could have caused the sample correlation (Cumming, 2014). * indicates $p < .05$. ** indicates $p < .01$.

SVS-GM3 = 3-item modified German Subjective Vitality Scale, SVS-GM1 = 1-item modified German Subjective Vitality Scale, ROF = Rate of Fatigue.

Supplementary Table 3

Means (M), standard deviations (SD), and Pearson product-moment correlation coefficients between the SVS-GM3, SVS-GM1, PA, NA and SWLS

Variable		*M*	*SD*	1	2	3	4	5	6	7	8	9	10	11	12	13	14
SVS-GM3	past (1)	6.19	2.23														
	present (2)	5.89	2.39	.70** [.60, .77]													
	future (3)	7.04	2.13	.69** [.59, .77]	.62** [.51, .72]												
SVS-GM1	past (4)	5.89	2.34	.80** [.74, .86]	.60** [.49, .70]	.58** [.46, .68]											
	present (5)	5.73	2.53	.54** [.42, .65]	.74** [.66, .81]	.52** [.39, .63]	.65** [.55, .74]										
	future (6)	6.78	2.29	.63** [.52, .72]	.63** [.52, .72]	.79** [.72, .84]	.63** [.52, .72]	.61** [.49, .70]									
PA	past (7)	3.24	0.65	.70** [.60, .77]	.53** [.40, .64]	.55** [.43, .66]	.71** [.62, .78]	.50** [.37, .61]	.45** [.31, .57]								
	present (8)	2.81	0.83	.48** [.35, .60]	.67** [.57, .75]	.48** [.34, .60]	.56** [.43, .66]	.72** [.64, .79]	.51** [.37, .62]	.64** [.53, .73]							
	future (9)	3.45	0.67	.45** [.31, .57]	.43** [.29, .56]	.67** [.57, .75]	.47** [.34, .59]	.49** [.35, .60]	.59** [.47, .69]	.64** [.53, .73]	.64** [.53, .72]						
NA	past (10)	1.94	0.58	-.47** [-.59, -.33]	-.33** [-.47, -.18]	-.37** [-.50, -.22]	-.39** [-.52, -.24]	-.26** [-.41, -.10]	-.29** [-.43, -.13]	-.31** [-.45, -.16]	-.26** [-.41, -.10]	-.23** [-.38, -.07]					
	present (11)	1.45	0.51	-.24** [-.39, -.08]	-.22** [-.37, -.06]	-.30** [-.44, -.15]	-.23** [-.38, -.07]	-.21* [-.36, -.05]	-.19* [-.34, -.02]	-.17* [-.32, -.01]	-.09 [-.25, .07]	-.20* [-.35, -.03]	.68** [.58, .76]				
	future (12)	1.60	0.54	-.37** [-.50, -.22]	-.30** [-.44, -.15]	-.47** [-.59, -.33]	-.32** [-.46, -.17]	-.25** [-.40, -.09]	-.42** [-.55, -.27]	-.22** [-.37, -.06]	-.28** [-.42, -.12]	-.40** [-.53, -.26]	.65** [.54, .73]	.53** [.41, .64]			

SWLS	past (13)	23.58	5.81	.64** [.53, .73]	.47** [.34, .59]	.52** [.39, .63]	.61** [.49, .70]	.37** [.22, .50]	.40** [.25, .53]	.60** [.48, .69]	.38** [.23, .51]	.35** [.20, .49]	-.48** [-.60, -.35]	-.23** [-.38, -.07]	-.26** [-.41, -.10]		
	present (14)	24.53	5.85	.53** [.40, .64]	.49** [.36, .61]	.47** [.33, .59]	.48** [.35, .60]	.41** [.26, .53]	.36** [.21, .49]	.47** [.33, .59]	.42** [.27, .55]	.34** [.19, .48]	-.47** [-.59, -.33]	-.33** [-.47, -.18]	-.28** [-.42, -.12]	.84** [.79, .89]	
	future (15)	25.17	5.29	.44** [.30, .56]	.42** [.28, .55]	.55** [.42, .65]	.40** [.25, .53]	.32** [.16, .46]	.46** [.32, .58]	.44** [.29, .56]	.33** [.17, .47]	.42** [.27, .55]	-.43** [-.56, -.29]	-.30** [-.44, -.14]	-.45** [-.57, -.30]	.72** [.63, .79]	.76** [.67, .82]

Note. N = 143

Values in square brackets indicate the 95% confidence interval for each correlation. The confidence interval is a plausible range of population correlations that could have caused the sample correlation (Cumming, 2014). * indicates $p < .05$. ** indicates $p < .01$.

SVS-GM3 = 3-item modified German Subjective Vitality Scale, SVS-GM1 = 1-item modified German Subjective Vitality Scale, PA = Positive Affect, NA = Negative Affect, SWLS = Satisfaction with Life Scale.

Supplementary Table 4

Means (M), standard deviations (SD), and Pearson product-moment correlation coefficients between the SVS-GM3, SVS-GM1, SWE, and LOT-R

Variable		*M*	*SD*	1	2	3	4	5	6	7
SVS-GM3	past (1)	6.31	2.19							
	present (2)	6.14	2.14	.62**<br>[.49, .73]						
	future (3)	6.82	2.11	.50**<br>[.34, .64]	.65**<br>[.52, .75]					
SVS-GM1	past (4)	6.12	2.41	.92**<br>[.88, .95]	.61**<br>[.48, .72]	.52**<br>[.36, .65]				
	present (5)	6.14	2.34	.57**<br>[.42, .69]	.88**<br>[.83, .92]	.60**<br>[.46, .71]	.59**<br>[.45, .71]			
	future (6)	6.63	2.26	.45**<br>[.28, .60]	.57**<br>[.42, .69]	.88**<br>[.83, .92]	.49**<br>[.32, .62]	.57**<br>[.42, .69]		
SWE	trait (7)	30.11	3.64	.51**<br>[.34, .64]	.50**<br>[.34, .64]	.44**<br>[.27, .59]	.50**<br>[.33, .63]	.45**<br>[.28, .59]	.44**<br>[.26, .58]	
LOT-R	trait (8)	22.56	3.62	.40**<br>[.22, .55]	.26**<br>[.07, .43]	.37**<br>[.19, .53]	.36**<br>[.17, .52]	.17<br>[-.03, .35]	.37**<br>[.19, .53]	.47**<br>[.30, .61]

Note. N = 100

Values in square brackets indicate the 95% confidence interval for each correlation. The confidence interval is a plausible range of population correlations that could have caused the sample correlation (Cumming, 2014). * indicates $p < .05$. ** indicates $p < .01$.

SVS-GM3 = 3-item modified German Subjective Vitality Scale, SVS-GM1 = 1-item modified German Subjective Vitality Scale, SWE = perceived self-efficacy, LOT-R = revised Life-Orientation Test.
