# Supplementary material for: The modified German subjective vitality scale (SVS-GM): Psychometric properties and application in daily life
Source: Front Psychol. 2022 Jul 29;13:948906. doi: 10.3389/fpsyg.2022.948906 (PMC9374102; doi:10.3389/fpsyg.2022.948906)
Supplement: SUPPLEMENTARY MATERIAL 3 — SM3_Buchner et al., SVS-GM Multilevel Analysis with the SVS-GM1 as the criterion measure. [file Table_3.pdf]

# Supplementary Material 3

## 1 Multilevel Analysis with the SVS-GM1 as the criterion measure

### 1.1 Data Analysis

We conducted an additional multilevel model analysis to examine daily and weekly fluctuation in subjective vitality with the SVS-GM1 as dependent variable. Because we were also interested in the relationship of fatigue and subjective vitality, we specified multilevel models with daily diary observations (Level 1) nested within participants (Level 2). In that model, we explored daily diary fluctuation (morning / noon / night), the influence of the type of day (workday vs. day off), and fatigue on subjective vitality. With the SVS-GM1 as the criterion measure and time of day, type of day and person mean centered ROF as predictors a random intercept model was significantly better than the null model (logLikelihood: -711.35, $p < .001$). Further a random slope for ROF fitted the data better than the random intercept model (logLikelihood: -705.49, $p = .003$).

## 2 Results

Subjective vitality was lowest in the morning when compared to noon and evening ($p < .001$). Subjective vitality was only significantly different on working days compared to days off, in simple model calculations, without ROF as a predictor. Furthermore, ROF predicted negatively subjective vitality ($p < .001$) with a different influence between subjects (slope). Results demonstrated within-person variation and between-person differences of the SVS-GM1 during the diary study. The results of the additional multilevel model analysis are presented in the Supplemantal Table 2.

### Supplementary Table 5

*Model Parameters for the additional Multilevel Analysis with the SVS-GM1 as the Criterion Measure*

| | | | 95% CI | |
|---|---|---|---|---|
| Parameter | Estimate (SE) | *p* | Lower | Upper |
| Fixed effects | | | | |
| Intercept | 5.89 (0.28) | <.001 | 5.34 | 6.44 |
| Noon vitality | 0.53 (0.12) | <.001 | 0.30 | 0.77 |
| Evening vitality | 0.71 (0.13) | <.001 | 0.46 | 0.96 |
| Day off | 0.21 (0.12) | 0.065 | -0.01 | 0.44 |
| ROF | -0.44 (0.04) | <.001 | -0.52 | -0.40 |
| Random effects | | | | |
| Level 2 (between-person) | | | | |
| Intercept | 1.81 | | 1.02 | 1.78 |
| ROF (Slope) | 0.02 | | 0.07 | 0.24 |
| Level 1 (within-person) | | | | |
| Residual | 1.05 | | 0.97 | 1.11 |

*Note*: Standard errors are in parentheses. All *p* values in this table are two-tailed.

SVS-GM1 = 1-item modified German Subjective Vitality Scale. The reference category for the variables noon vitality and evening vitality is morning vitality.
